# Metabolite Identification of Isopropoxy Benzene Guanidine in Rat Liver Microsomes by Using UHPLC-Q-TOF-MS/MS

**DOI:** 10.3390/ijms24087313

**Published:** 2023-04-15

**Authors:** Yixing Lu, Wanying Zhang, Yongxiang Zhang, Sujuan Wu, Minglang Ma, Xianfeng Peng, Zhenling Zeng, Dongping Zeng

**Affiliations:** 1 Guangdong Provincial Key Laboratory of Veterinary Pharmaceutics Development and Safety Evaluation, College of Veterinary Medicine, South China Agricultural University, Guangzhou 510642, China; lyx2021@stu.scau.edu.cn (Y.L.); 20212027048@stu.scau.edu.cn (W.Z.); yongxiang@stu.scau.edu.cn (Y.Z.); wusujuan@stu.scau.edu.cn (S.W.); minglang@stu.scau.edu.cn (M.M.); 2 National Risk Assessment Laboratory for Antimicrobial Resistance of Animal Original Bacteria, Guangzhou 510642, China; 3 Guangzhou Insighter Biotechnology Co., Ltd., Guangzhou 510663, China; pengist@hotmail.com

**Keywords:** isopropoxy benzene guanidine, metabolites, liver microsomes, UHPLC-Q-TOF-MS/MS

## Abstract

Isopropoxy benzene guanidine (IBG) is a guanidine derivative with antibacterial activity against multidrug-resistant bacteria. A few studies have revealed the metabolism of IBG in animals. The aim of the current study was to identify potential metabolic pathways and metabolites of IBG. The detection and characterization of metabolites were performed with high-performance liquid chromatography tandem mass spectrometry (UHPLC-Q-TOF-MS/MS). Seven metabolites were identified from the microsomal incubated samples by using the UHPLC-Q-TOF-MS/MS system. The metabolic pathways of IBG in the rat liver microsomes involved O-dealkylation, oxygenation, cyclization, and hydrolysis. Hydroxylation was the main metabolic pathway of IBG in the liver microsomes. This research investigated the in vitro metabolism of IBG to provide a basis for the further pharmacology and toxicology of this compound.

## 1. Introduction

Antibiotic resistance poses a serious threat to public health and modern healthcare [1]. ESKAPE (*Enterococcus faecium*, *Staphylococcus aureus*, *Klebsiella pneumoniae*, *Acinetobacter baumannii*, *Pseudomonas aeruginosa*, and *Enterobacter* species) pathogens are known for their resistance to conventional antibiotics, which greatly increases the failure rate of antimicrobial treatment [2,3]. These pathogens exhibit resistance to first-line and final-resort antibiotics worldwide, leading to the failure of existing drugs [4,5]. Thus, new drugs must be developed to control infections from drug-resistant bacteria [6,7].

In medicinal chemistry, guanidine compounds are promising candidates for further structural modification of drugs due to their strong organic base and hydrophilicity, and some of them have been widely used to treat many diseases [8,9]. Guanidine-containing compounds also have good antibacterial activity against drug-resistant bacteria [10,11]. Isopropoxy benzene guanidine (IBG) is a novel guanidine compound that not only exhibits effective antibacterial activity against Gram-positive bacteria but also restores the sensitivity of Gram-negative bacteria to colistin as an antibiotic adjuvant [12,13,14,15]. IBG is expected to be a new antibacterial drug for bacterial infections. Drug metabolism plays a vital role in pharmacokinetics and drug safety evaluation because some metabolites may have pharmacological activity, reactivity, or toxicity [16,17]. Therefore, the investigation of the metabolic fate of candidate compounds is an important part of new drug research and development [18]. However, the identification and profiling of IBG metabolites has not been reported to date. 

Two common approaches, namely, in vitro and in vivo, are utilized to study drug metabolism. In vitro methods involve incubating the drug with multiple types of tissue components, including liver microsomes and perfused hepatocytes, isolated from experimental animals [19,20]. Liver microsomes make up a common in vitro system for evaluating drug metabolism profiles [21,22]. Liquid chromatography/tandem mass spectrometry has become a good option for the identification and structural characterization of metabolites due to its selectivity, sensitivity, and fast analytical speed [23,24]. Mass spectrometry is also widely used to detect and identify drug metabolites in vitro and in vivo [25,26].

Evidently, the rapid and accurate detection and identification of the metabolites of IBG are crucial. Quadrupole time-of-flight mass spectrometry, with its high mass resolution and accuracy, is considered a good choice for identifying and characterizing metabolites [27,28]. This present study investigated the in vitro metabolism of IBG in rat liver microsomes. For this purpose, an ultrahigh-performance liquid chromatography quadrupole time-of-flight mass spectrometry system (UHPLC-Q-TOF-MS/MS) was established to characterize the formed metabolites. We established the chemical structures of the detected metabolites on the basis of accurate MS2 spectra and elemental compositions. 

## 2. Results

### 2.1. SOM Prediction Using SMARTCyp

**Figure 1:**
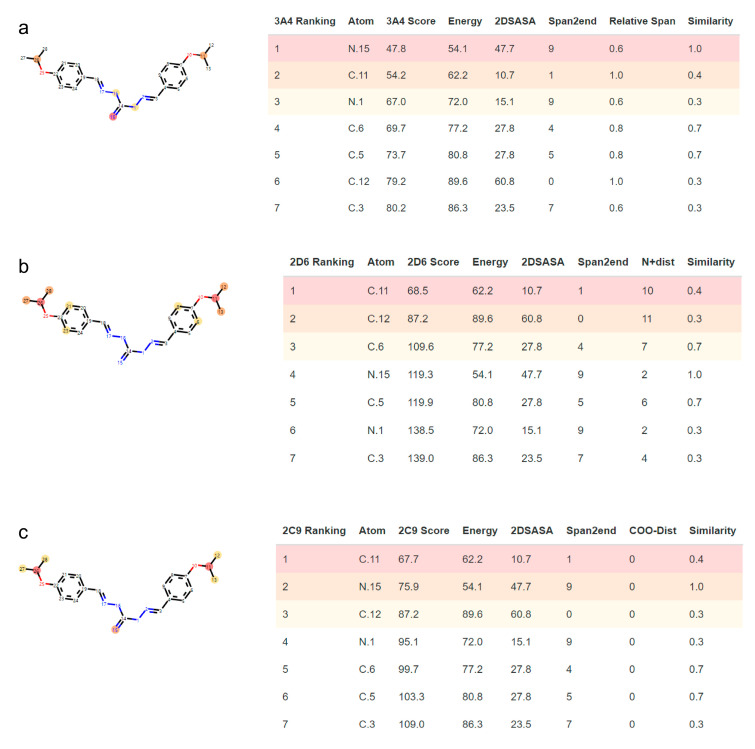
Metabolic site prediction using SMARTCyp web server by CYP3A4 (**a**), CYP2D6 (**b**), and CYP2C9 (**c**) for IBG.

Figure 1 shows the results of the IBG molecule sites most liable to metabolism by CYP450. The SMARTCyp standard algorithm (CYP3A4) demonstrated that the potential IBG sites of fragmentation were N.15 site (Score = 47.8; Energy = 54.1), C.11 site (Score = 54.2; Energy = 62.2), and N.1 site (Score = 67.0; Energy = 72.0). The SMARTCyp CYP2D6 algorithm indicated that the potential IBG sites of fragmentation were C.11 site (Score = 68.5; Energy = 62.2), C.12 site (Score = 87.2; Energy = 89.6), and C.6 site (Score = 109.6; Energy = 77.2). The SMARTCyp CYP2C9 algorithm highlighted that the potential IBG sites of fragmentation were C.11 site (Score = 67.7; Energy = 62.2), N.15 site (Score = 75.9; Energy = 54.1), and C.12 site (Score = 87.2; Energy = 89.6). The lowest score with the CYP3A4 algorithm were C.3 (Score = 80.2; Energy = 86.3), C.12 (Score = 79.2; Energy = 89.6), and C.5 (Score = 73.7; Energy = 80.8). With applying the SMARTCyp CYP2D6 algorithm, the most discouraged fragmentations were C.3 (Score = 139.0; Energy = 86.3), N.1 (Score =138.5; Energy = 72.0), and C.5 (Score = 119.9; Energy = 80.8). Based on the SMARTCyp CYP2C9 algorithm, the least recommended fragmentations were C.3 (Score = 109.0; Energy = 86.3), C.5 (Score = 103.3; Energy = 80.8), and C.6 (Score = 99.7; Energy = 77.2). The fragment energy at O.10, O.25, C.7, C.22, N.2, N.17, C.14, C.4, and C.19 sites were without the matching energy rule (energy equal to 999 with each algorithm applied). Based on the three different SMARTCyp algorithms, the primary SOM in IBG was C.11 of the propyl group attached to the ether bond. The secondary predicted SOM was N.15 of the guanidine group, while the tertiary SOM was C.12 of the propyl group.

### 2.2. Mass Spectrometric Analysis of IBG

**Figure 2:**
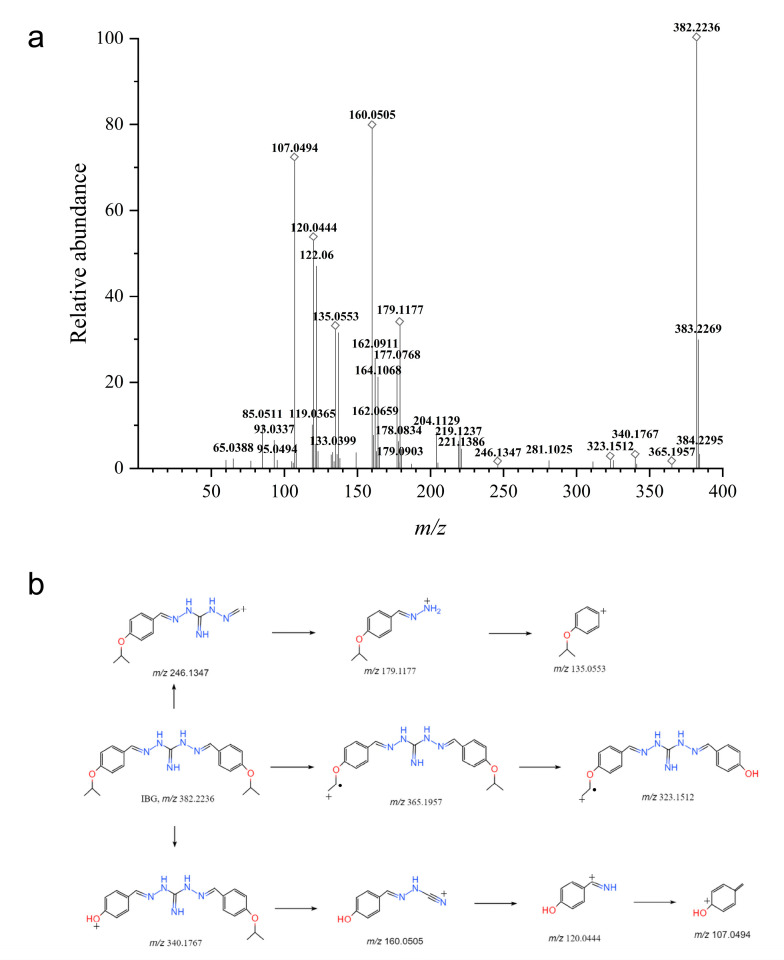
Collision-induced dissociation spectrum of IBG (**a**) and proposed fragmentations (**b**).

The parent compound IBG was analyzed using UHPLC-Q-TOF-MS/MS. The precursor ion [M+H]^+^ of IBG was detected at a retention time of 11.43 min with *m*/*z* 382.2336 (0.38 ppm, elemental composition C_17_H_28_N_5_O_2_^+^). In the product ion scan mode, the major fragment ions were acquired at *m*/*z* 107.0494, 120.0444, 135.0553, 160.0505, 179.1177, and 323.1512 (Figure 2a). The possible cleavage pathways of IBG are shown in Figure 2b. The fragment ion at *m*/*z* 246.1347 was formed by the loss of isopropoxybenzene (-C_9_H_12_O). This ion further produced the ions at *m*/*z* 179.1177 through the loss of (2-methylidenediazanyl) methanimine (-C_2_H_5_N_3_). The fragment at *m*/*z* 160.0505 was attributed to the protonated methanimidoyl phenol moiety in IBG. The fragment ion at *m*/*z* 135.0553 was formed by isopropoxybenzene (C_9_H_12_O). The fragment ion at *m*/*z* 120.0444 was attributed to 4-(azanylidenemethyl) phenol (C_7_H_7_NO). The fragment ion at *m*/*z* 107.0494 was attributed to 4-methylphenol(C_7_H_8_O). 

### 2.3. Mass Spectrometric Analysis of the Microsomal Incubation Samples 

**Figure 3:**
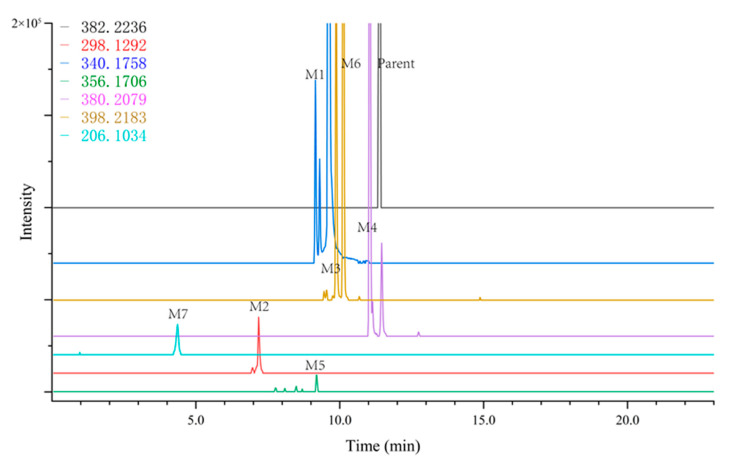
Extracted mass chromatograms of IBG metabolites in rat liver microsomes incubated with IBG for 1.5 h.

The metabolites were identified by comparing the samples with the blank via total ion flow chromatography. The ion chromatograms extracted from total ion flow chromatography confirmed the presence of probable metabolites. Figure 3 shows the detailed extracted ion chromatograms for the ions at *m*/*z* 206.1034, 298.1292, 340.1758, 356.1706, 380.2079, and 398.2183 from the full scan of IBG incubated with rat liver microsomes. These were assumed to be IBG metabolites on the basis of the comparison of incubated samples with control samples and the agreement of accurate mass measurement in MS spectra with the prediction formula calculations (within 10 ppm). The predicted elemental compositions, exact measured mass, and mass error of the metabolites are shown in Table 1. The accurate and measured masses were consistently below 5 ppm, thereby supporting the proposed elemental composition of the metabolites. 

### 2.4. Structural Elucidation of Metabolites

**Figure 4:**
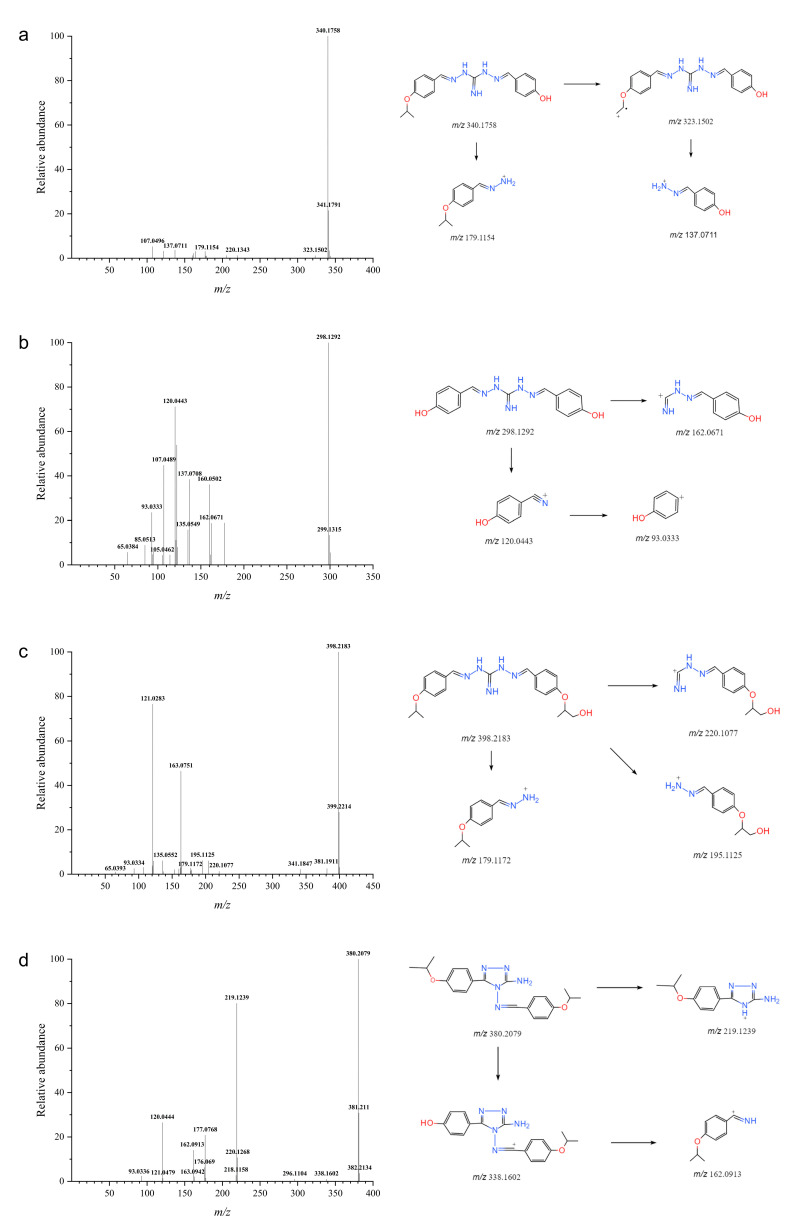
Collision-induced dissociation spectra of M1 (**a**), M2 (**b**), M3 (**c**) and M4 (**d**) and their proposed fragmentations.

M1 was detected at a retention time of 9.15 min. The protonated molecular ion [M+H]^+^ at *m*/*z* 340.1758 was 42 Da lower than IBG, suggesting that M1 is an O-dealkylation product of IBG. The fragmented mass data (Figure 4a) showed two indicative fragment ions at *m*/*z* 179.1154 and 323.1512, which were formed by the oxidation of the one-sided isopropoxy group. The fragment ions at *m*/*z* 107, 122, and 179 remained unaffected, similar to those in the parent ion MS/MS spectrum. 

M2 was eluted at a retention time of 7.19 min and the protonated molecular ion [M+H]^+^ was observed at *m*/*z* 298.1292, 42 Da lower than that for M1, indicating that M2 was O-dealkylation product of M1. Two indicative fragment ions at *m*/*z* 120.0443 and 162.0671, further confirming the loss of the isopropoxy group (Figure 4b). 

M3 was detected at a retention time of 9.88 min, and the protonated molecular ion [M+H]^+^ was observed at *m*/*z* 398.2183. A higher mass shift of 16 Da was observed between masses of M3 compared to IBG, indicating that M3 was an oxidation product of the IBG. The MS/MS spectrum (Figure 4c) displayed an informative fragment ion at *m*/*z* 195.1125, which further confirmed the occurrence of methyl oxidation. Moreover, the appearance of *m*/*z* 179.1172 fragment ions in the fragmented mass data of M3 suggested the presence of isopropoxy in M3.

M4 was eluted at a retention time of 11.45 min. The protonated molecular ion [M+H]^+^ was detected at *m*/*z* 380.2079. Moreover, the presence of fragment ions at *m*/*z* 380 (-H2 from *m*/*z* 382), *m*/*z* 338 (-C_3_H_6_N from *m*/*z* 380), *m*/*z* 219 (-C_10_H_11_NO from *m*/*z* 380), and *m*/*z* 162 (-C_8_H_6_N_4_O from *m*/*z* 338) further supported the formation of M4 (Figure 4d). 

**Figure 5:**
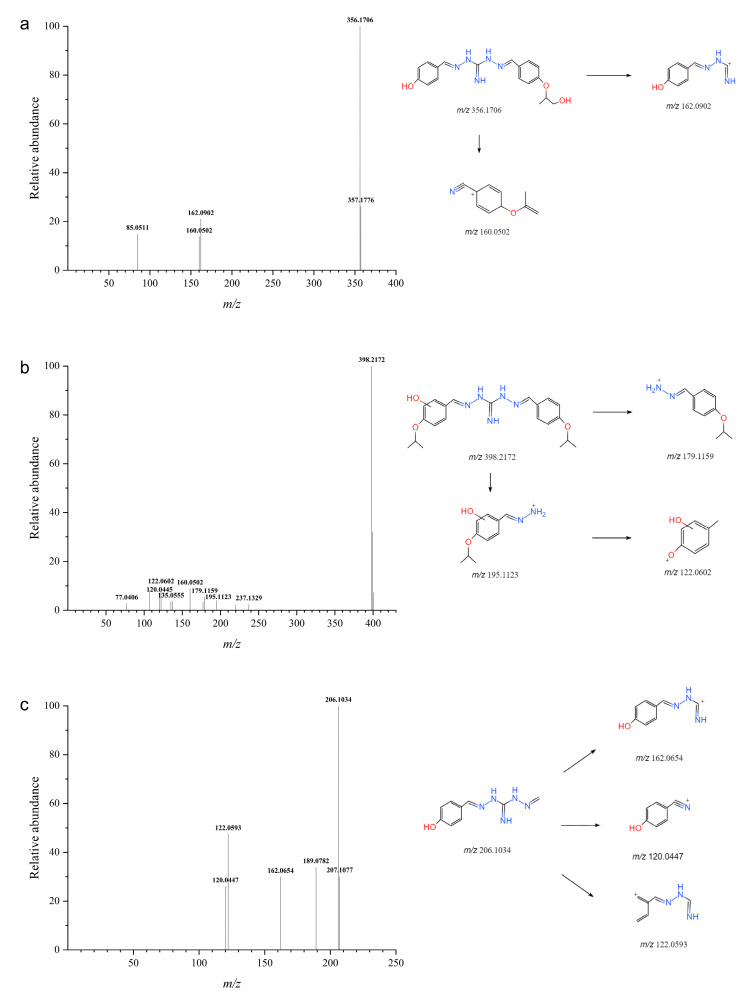
Collision-induced dissociation spectra of M5 (**a**), M6 (**b**), and M7 (**c**), along with their proposed fragmentations.

M5 was eluted at a retention time of 8.49 min, and the protonated molecular ion [M+H]^+^ was observed at *m*/*z* 356.1706, which was 42 Da lower than that for M3, indicating that M5 was separated from M3 by oxygenation. The MS/MS spectrum revealed an indicative fragment ion at *m*/*z* 160.0502 and 162.0902, which was formed by the dealkylation of isopropoxy and the loss of isopropoxy (Figure 5a).

M6 was detected at a retention time of 10.11 min. The protonated molecular ion [M+H]^+^ was observed at *m*/*z* 398.2172, indicating that M6 was a hydroxyl metabolite on the isopropoxybenzene ring. The MS/MS spectrum (Figure 5b) showed an informative fragment ion at *m*/*z* 195.1123, 179.1125 and 122.0602, which further confirmed the hydroxylation on one side of the benzene ring. The metabolism may occur in one of the carbon atoms of the benzene ring.

M7 had a retention time of 4.36 min, an [M+H]^+^ ion at *m*/*z* 206.1034, and an elemental composition of C_9_H_12_N_5_O^+^, indicating that it was a hydrolyzed metabolite of M2. The MS/MS spectrum (Figure 5c) of M7 displayed a fragment ion at *m*/*z* 162.0654 identical to M2.

## 3. Discussion

Compounds containing the guanidine group constitute a class of bioactive molecules with a wide range of applications [10,29]. Thus, guanidine has been developed as the dominant structural motif in the design of novel drugs for the treatment of various infectious diseases [30,31]. IBG is a substituted benzoguanidine derivative with good antibacterial activity against Gram-positive and Gram-negative bacteria [13,14,15]. The purpose of this work was to study the metabolism of IBG for the first time by using in vitro methods and analytical tools. The enzymes involved in the drug metabolism were mainly CYP450 [32]. Since most members of the CYP450 family members were primarily expressed in the liver, the in vitro liver system was the most commonly used biotransformation model when drug metabolism was considered [33]. Liver microsomes were a common in vitro system for assessing drug metabolism profiles [34]. We used sensitive and specific UHPLC-Q-TOF-MS/MS to analyze the structure of IBG metabolites. The structures of the detected IBG metabolites were characterized by the mass variation of the parent drug or metabolite, the molecular formula obtained from accurate mass measurements, and accurate MS/MS spectral interpretation.

During drug development, it is important to identify the metabolic site of the parent compound at an early stage to guide the development of a compound with an ideal pharmacokinetic profile [35]. The SMARTCyp application was used to obtain metabolic prediction sites for CYP450-mediated IBG metabolism [36]. The analysis of three different SMARTCyp algorithms revealed that IBG has five potential sites in the molecular structure. The most active molecular fragment appears to be at position C.11. In the case of IBG metabolites, the hydroxylation of M1, M2, and M7 occurred at the corresponding positions. The in vitro culture of rat liver microsomes confirmed the predicted results. However, no reaction was found with N.1 and N.15 in the seven metabolites of IBG. Combined with the prediction results of SMARTCyp and the metabolic study of rat liver microsomes, CYP3A4, CYP2D6, and CYP2C9 may be involved in the metabolic disposal process of IBG.

**Figure 6:**
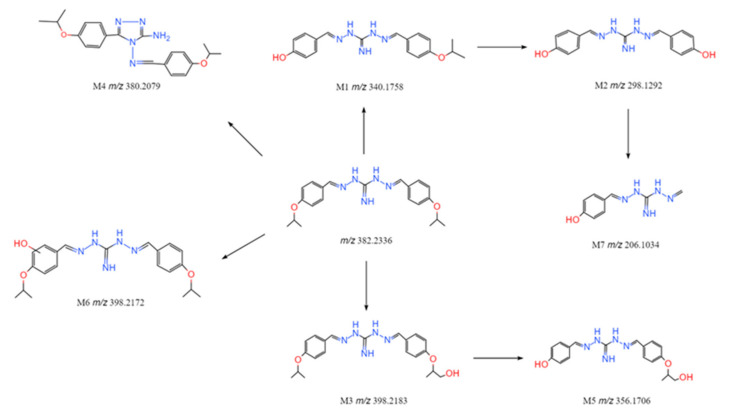
Proposed in vitro metabolic pathways of IBG after exposure to rat liver microsomes.

Through UHPLC-Q-TOF-MS/MS, a total of seven metabolites were extracted from rat liver microsomes exposed to IBG. Figure 6 shows the possible metabolic pathways of IBG in the rat liver microsomes. Metabolites M1, M3, M4, and M6 were the primary metabolites, and M2, M5, and M7 were the secondary metabolites. The metabolic pathways of IBG in the rat liver microsomes involved O-dealkylation (M1, M2, M5, M7), oxygenation (M3, M5, M6), cyclization (M4), and hydrolysis (M7). The identification of five hydroxylated metabolites of IBG revealed that hydroxylation is the major pathway for IBG in rat liver microsomes. Cyclization was a common metabolic pathway in guanidine-containing compounds. Metabolite M4 was formed through a cyclization reaction, which was also present in the metabolite of the anticoccidized drug robenidine [37]. Proguanil was a widely used antimalarial drug, which metabolized by CYP2C19 to the main active metabolite cycloguanil [38]. The identification of IBG metabolites in liver microsomes lays a foundation for further research on the metabolism of this compound in vivo.

## 4. Materials and Methods

### 4.1. Materials and Instruments

IBG (99.9%) was obtained from Guangzhou Insighter Biotechnology (Guangzhou, China). Sprague–Dawley rat liver microsomes and a Phase I metabolic stability kit was purchased from IPHASE Co., Ltd. (Beijing, China, Lot: 111011). Acetonitrile and formic acid of HPLC-grade were bought from Sigma-Aldrich (Oakville, ON, Canada). Deionized water was prepared using a Milli-Q purification system (Millipore Corp., Bedford, MA, USA). An LC-QTOF-MS/MS system equipped with an Agilent 1290 system (Agilent Technologies, Santa Clara, CA, USA) and a 6540 UHD QTOF mass spectrometer (Agilent Technologies, USA) was employed.

### 4.2. SMARTCyp Prediction of IBG Molecule Sites

The sites of metabolism prediction of cytochrome P450-mediated drug metabolism were obtained using the SMARTCyp server web application (www.farma.ku.dk/smartcyp, accessed on 3 December 2022) [36,39,40]. 

### 4.3. In Vitro Metabolism of IBG by Rat Liver Microsomes

The liver microsomes (0.5 mg/mL) were suspended in a NADPH-generating system with a 0.1 M phosphate buffer (pH 7.4). There was a total volume of 200 μL for incubation. IBG (50 μM) was added to the incubations to initiate the reactions after the mixtures were preincubated for 5 min at 37 °C. Incubations without IBG, liver microsomes, or NADPH were regarded as controls. Less than 0.5% (*v*/*v*) of the organic solvent was present in the total volume. The reaction was terminated with the addition of 200 μL of ice-cold acetonitrile after they had been incubated at 37 °C for 90 min. Then the samples were vortexed and centrifuged at 16,000× *g* for 10 min. The supernatant was filtered with a 0.22 μm filter, and aliquots were analyzed using UHPLC-Q-TOF-MS/MS for the identification of metabolites. 

### 4.4. Instrument and Analytical Conditions

The established UHPLC-Q-TOF-MS/MS was composed of an Agilent 1290 system (Agilent Technologies, USA) and a 6540 UHD QTOF (Agilent Technologies, USA) equipped with an electrospray ionization source (ESI) and operated in positive ion mode. The separation was carried out on an Eclipse Plus C18 column (100 mm × 2.1 mm; 1.8 μm; Agilent) with 0.2% formic acid (solvent A) and acetonitrile (solvent B). Gradient eluting was carried out at 0.3 mL/min. The gradient conditions were optimized as follows: 5% B at 0–1 min, 5–20% B at 1–5 min, 20–50% B at 5–10 min, 50–90% B at 10–15 min, 90% B at 15–18 min, 90–5% B at 18–18.1 min, and 5% B at 18.1–23 min. The injection volume was 10 μl. Autosamplers were set to 4 °C for sample chambers and 40 °C for columns. 

At a scan rate of one spectrum per second, Q-TOF-MS was ran in ESI positive mode scanning (*m*/*z*) from 50 amu to 1000 amu. For MS, the following parameters were used: 300 °C gas temperature, 8 L/min flow rate, 45 psi nebulizer pressure, 350 °C sheath gas temperature, 10 L/min flow rate. Voltages of 1000, 4000, 100, and 65 V were applied to the nozzle, capillary, fragmentor, and skimmer, respectively. The instrument was calibrated during the run times by monitoring positive ions at reference masses *m*/*z* 121.0508 and 922.0097.

In the mass spectrometry process, the molecular formula search technique was used to obtain the information of the target compounds, and then the molecular feature search technique was used to search the compounds by element composition in the range of 50-1000 molecular weight. The selected candidate compounds were used as the parent ion setting for the secondary mass spectrometry analysis. MassHunter Qualitative Analysis B.08.00 (Agilent Technologies, USA) was used to evaluate all MS data. The formula predictor also calculated all masses corresponding to a specific elemental composition and generated multiple formulas suggested by the software. An accuracy error threshold of ±10 ppm was used as a limit for calculating possible elemental compositions. The Fragmentation Library in MassFrontier6.0 (Thermo Fisher Scientific, Waltham, MA, USA) was used to predict fragmentation according to the compound structure.

## Figures and Tables

**Table 1:** Summary of IBG metabolites in rat liver microsomes.

Metabolites	RT (min)	Metabolic Pathway	Elemental Composition ([M+H]^+^)	Measured Mass (*m*/*z*)	Theoretical Mass (*m*/*z*)	Mass Error (ppm)
M1	9.15	O-dealkylation	C_18_H_22_N_5_O_2_^+^	340.1758	340.1768	2.93
M2	7.19	O-dealkylation	C_15_H_16_N_5_O_2_^+^	298.1292	298.1299	2.34
M3	9.88	Oxygenation	C_21_H_28_N_5_O_3_^+^	398.2183	398.2187	1.00
M4	11.45	Cyclization	C_21_H_26_N_5_O_2_^+^	380.2079	380.2081	0.52
M5	9.16	O-dealkylation and oxygenation	C_18_H_22_N_5_O_3_^+^	356.1706	356.1717	3.08
M6	10.11	Oxygenation	C_21_H_28_N_5_O_3_^+^	398.2172	398.2187	3.76
M7	4.36	O-dealkylation and hydrolysis	C_9_H_12_N_5_O^+^	206.1034	206.1036	0.97
Parent	11.43	Parent	C_21_H_28_N_5_O_2_^+^	382.2236	382.2238	0.52

## Data Availability

No new data were created or analyzed in this study. Data sharing is not applicable to this article.
